# Opportunities and Challenges for Smartphone Applications in Supporting Health Behavior Change: Qualitative Study

**DOI:** 10.2196/jmir.2583

**Published:** 2013-04-18

**Authors:** Laura Dennison, Leanne Morrison, Gemma Conway, Lucy Yardley

**Affiliations:** ^1^Academic Unit of PsychologyUniversity of SouthamptonSouthamptonUnited Kingdom

**Keywords:** mobile phone, cellular phone, behavior, health, qualitative research, focus groups

## Abstract

**Background:**

There is increasing interest from academics and clinicians in harnessing smartphone applications (apps) as a means of delivering behavioral interventions for health. Despite the growing availability of a range of health-related apps on the market, academic research on the development and evaluation of such apps is in the relatively early stages. A few existing studies have explored the views of various populations on using mobile phones for health-related issues and some studies are beginning to report user feedback on specific apps. However, there remains little in depth research on users’ (and potential users’) experiences and views on a wide range of features and technologies that apps are, or will soon be, capable of. In particular, research on young adults is lacking, which is an unfortunate omission considering that this group comprises of a good number of mobile technology adoptors.

**Objective:**

The current study sought to explore young adults’ perspectives on apps related to health behavior change. It sought their experiences and views of features that might support health behavior change and issues that contribute to interest in and willingness to use such apps.

**Methods:**

Four focus groups were conducted with 19 students and staff at a University in the United Kingdom. Participants included 13 females and 6 males with a mean age of 23.79 (SD 7.89). The focus group discussions centred on participants’ experiences of using smartphone apps to support a healthy lifestyle, and their interest in and feelings about features and capabilities of such apps. The focus groups were recorded, transcribed, and analyzed using inductive thematic analysis.

**Results:**

Study findings suggested that young, currently healthy adults have some interest in apps that attempt to support health-related behavior change. Accuracy and legitimacy, security, effort required, and immediate effects on mood emerged as important influences on app usage. The ability to record and track behavior and goals and the ability to acquire advice and information “on the go” were valued. Context-sensing capabilities and social media features tended to be considered unnecessary and off-putting.

**Conclusions:**

This study provided insight into the opportunities and challenges involved in delivering health-related behavioral interventions through smartphone apps. The findings suggested a number of valued features and characteristics that app developers may wish to consider when creating health behavior apps. Findings also highlighted several major challenges that appeared to need further consideration and research to ensure the development of effective and well-accepted behavior change apps.

## Introduction

### Background

The smartphone market is growing rapidly with statistics from 2011 and 2012 suggesting that 35% of US and 39% of UK adults, respectively, use smartphones, with acceptance increasing quickly [1,2]. These ubiquitous devices are increasingly complex, computationally powerful, sensor-rich, and integrated with social networking [3-5]. Alongside these technological developments, there has been increasing interest from academics and clinicians in harnessing smartphones as a means of delivering behavioral interventions for health.

Various features of smartphones make them good candidates for the delivery of behavioral interventions. First, as portable devices that are highly valued by individuals, they tend to be switched on and remain with the owner throughout the day [3,5,6]. Therefore, they offer the opportunity to bring behavioral interventions into important real life contexts where people make decisions about their health and encounter barriers to behavior change [4,5,7]. Second, smartphone apps may provide cheaper, more convenient, or less stigmatizing interventions that are unavailable elsewhere [4,7]. Third, the connectedness of smartphones facilitates the sharing of behavioral and health data with health professionals or peers [4,6]. Furthermore, the increasing ability of smartphones to use internal sensors to infer context such as user location, movement, emotion, and social engagement (eg, [8-10]) has raised the prospect of continuous and automated tracking of health-related behaviors and timely, tailored interventions for specific contexts.

In addition to the existing body of research on telephone and SMS (short message service) text-messaging-delivered interventions, smartphone software programs, or applications (apps) have stimulated significant attention in recent years. For example, many thousands of commercial apps have already been developed to assist people with managing stress, improving mood, following a healthy diet, increasing physical activity, quitting smoking, and self-managing chronic health problems. Apps aiming to improve health tend to provide information, advice, instruction, prompts, support, encouragement, and interactive tools for individuals to monitor, record, and reflect.

Although there has been much enthusiasm for delivering interventions through smartphone apps, academic research on the development and evaluation of such apps is in the early stages. At this juncture, as well as initial pilot studies of specific individual apps, there is a need for formative research that helps us to better appreciate the interest various groups of people have in using these sorts of apps and factors that may influence acceptability and engagement.

### Previous Studies and Interventions

Some initial qualitative research on mobile phone use for health has already been conducted with various populations in several contexts including both healthy [11,12] and chronically ill [11] adults, and adults at risk of developing lifestyle-related health conditions [13]. Findings to date indicated that using mobile phones for managing physical and mental health and for supporting changes in health-related behavior is acceptable in principle to many people. A number of studies have focused more specifically on experiences of smartphone apps. Some such studies have been nested within pilot studies of research-developed apps. For instance, a feasibility study in 8 users of a mobile intervention for depression that included ecological momentary intervention and context sensing found that participants were satisfied with the intervention, despite encountering substantial technical problems [14]. Other studies have concentrated on understanding useful features of one or more specific apps. In one study, 15 participants downloaded 3 physical activity apps, which they used for one week each. They then provided quantitative ratings of the apps and qualitative feedback about their experiences. Users preferred flexible apps with automatic tracking of physical activity, tracking of goal progress, music features, and well-documented and easy to use features [15]. Other research, nested within an intervention trial, collected data on 119 users’ experiences of 3 different apps (a wellness diary, a physical activity coach, and a relaxation assistant) and generated detailed findings relating to motivators and barriers to using each app [16].

Overall, the literature on smartphone app feasibility and acceptability is encouraging. However, in previous research, the exploration of user viewpoints has often been limited and fairly superficial. There is little in depth, qualitative research allowing users to describe their experiences, views, and usage patterns. Several important areas are inadequately addressed by previous research. First, there is an absence of research on how young adults perceive and use apps for behavior change. Few reports exist discussing the development of interventions for this population [17] despite the tendency for young adults to be early adoptors of smartphone technology [2,17]. Recent publications have highlighted the lack of focus on smartphone interventions for young adults and suggested that this is a priority population for a broad range of health behavior issues [17,18]. Second, tens of thousands of health apps are available directly to consumers through the market (eg, Apple App Store, Google Play) and a 2011 report showed that 11% of US adult smartphone users download health apps [19]. Reviews suggested that most direct-to-consumer apps are not developed by health professionals or academics, do not draw on behavior change theories or techniques, and do not have content aligned to clinical guidelines for the condition or behavior in question [20-24]. However, there is a surprising paucity of academic research on user views and experiences of these apps. Finally, there has been little exploration of people’s views about context sensing by smartphones in the setting of health apps, although it has been recognized that this advancing technology may raise concerns, including worries around regarding privacy and security [4,25].

### Current Study

The current study sought to explore some of these under-researched areas. We conducted a series of focus groups with healthy young adult smartphone users in order to explore: (1) their existing experiences of using health-related smartphone apps, and (2) their views about a range of different features, technologies, and capabilities that characterize currently available or future apps. We sought their views on features that might support them in making changes to behaviors relevant to health, factors that contribute to interest in apps, willingness to use the apps, and issues leading to disinclination to using the apps.

## Methods

### Participants

A total of 19 participants were recruited through advertisements placed around Southampton University campus. Eligible participants were 18 years of age or older, and owned a smartphone. Prior to arrival at the focus group, all participants completed a brief online questionnaire providing demographic, lifestyle, and smartphone-related data. Participants comprised of 13 females, 6 males, 16 students, and 3 junior members of staff. The age range was 18 to 50 (mean 23.79, SD 7.89) and 16/19 (84%) were White British. At the time of the study, 10 participants owned iPhones, 5 owned Android phones, and 4 owned Blackberries. All participants reported that they had previously used smartphone apps. The participants were relatively frequent users of their phones with all but two reporting at least one hour of use per day. Sixteen reported using non-call features (eg, apps, camera, Internet, multimedia) “often” or “very often” and only one used these “rarely”. Fifteen participants indicated that they had previously sought health-related advice or information from the Internet or other forms of technology. Participants rated themselves as relatively “up to date with the latest technology” (minimum 3, mean 3.84, SD 6.02 on a 1-5 Likert scale). In terms of health and lifestyle, 7/19 (37%) participants reported themselves as “overweight”, whereas 12/19 (63%) classified themselves as “about right”. Around half (10/19, 53%) reported that they “lead an active lifestyle”. Most (14/19, 74%) believed themselves to “eat a healthy diet”.

### Procedure

Four focus groups were conducted (with 3, 5, 5, and 6 participants in each group). The session was guided by an interview schedule (Multimedia Appendix 1). The discussion began with questions around participants’ personal experiences of smartphone apps for health. To prompt further discussion, particularly among participants with limited experience of relevant apps, the facilitator presented trigger materials. Images showing features and capabilities of health-related apps were displayed on a laptop computer using PowerPoint. This included examples of apps that: (1) assisted with setting goals and reviewing progress towards goals, (2) gave advice, tips, and information on health, (3) used tools to monitor behavior, mood, and well-being, (4) issued reminders and prompts for healthy behaviors, (5) allowed the sharing of progress through online social media, and (6) made use of context sensing to issue prompts or interventions (Multimedia Appendix 2). Participants were then asked to describe their thoughts and feelings about the features of these types of apps, including their perceived usefulness, relevance, and concerns. Participants themselves tended to guide the conversation, while the facilitator provided prompts to pursue more detailed discussions on some topics and to keep the discussions on track.

The focus groups were 55 to 70 minutes. Two researchers participated in each group—the first author (a post-doctoral researcher in health psychology) and the third author (a psychology student trained in focus group data collection methods). In each group, one researcher facilitated the discussion and presented the trigger materials, while the other assisted and took notes.

### Data Analysis

The focus groups were audio recorded and transcribed verbatim. The transcripts were then analyzed using inductive thematic analysis [26]. Following initial coding, where labels were attached to text segments which appeared to indicate important material in relation to the research questions, analysis progressed in an iterative fashion to develop a set of themes that captured the essence of the focus group discussions. The analyst compared the raw data with the emerging theme labels and definitions, and further refined the themes by merging, adding, and removing redundant themes.

## Results

### Overview

Many participants in this study were positive about the potential for phones to help people adopt healthier lifestyles. Most had already discovered and tried various apps to support healthy lifestyle practices. Many female participants had used calorie counting apps to help manage their weight. The male participants tended to describe using apps that supported physical fitness. Many participants expressed interest in trying the apps discussed within the focus groups. Despite appearing to be a health-conscious and enthusiastic group, the participants nonetheless perceived considerable barriers, concerns, and opportunities related to health apps. Table 1 summarizes the key themes identified from the focus groups. The following sections describe the themes with illustrative quotations. Pseudonyms have been used to preserve anonymity.

**Table 1:** Overview of themes.

Theme	Summary of key points identified
Smartphones as valuable information sources	Smartphones are valued for providing a quick and efficient link to a wealth of information “on the go”.
Tracking progress and raising awareness	Apps that support monitoring, tracking, and reviewing of behavior are interesting and potentially useful. Tracking progress must be both accurate and require little effort. Tracking features have the potential to trigger negative emotions.
Behavior change apps and social networks	A social undesirability of using health-related apps exists. There is an aversion to sharing information related to health behavior via online social networks (except in circumscribed contexts).
Scepticism over context sensing	Context sensing can be perceived as “gimmicky”, unreliable, and unnecessary. Interventions triggered by context sensing could have negative consequences including irritation, poor mood, and more unhealthy behavior.
Useful prompts or harassment?	Prompts or reminders from apps can be useful, but can also become annoying and perceived as “nagging”
Motivation and necessity of behavior change	The appeal and usefulness of apps and their features is dependent on users’ existing intentions and motivation to change. Currently healthy young adults may perceive heath apps as of limited relevance to themselves and more suitable for people with “more serious” need for behavior change interventions (eg, people with existing health problems)
Necessity for efficiency and convenience	Smartphones are expected to be efficient and pleasurable to interact with. Health apps are often perceived as time-consuming or burdensome and thus unlikely to be used for extended periods of time.
Disposability	There are plenty of free apps to choose from with little commitment to long-term use of any particular app. Apps are easily downloaded and just as easily uninstalled.
Credibility and accuracy	Reputable and legitimate sources are considered important. Safe and accurate app content is a concern to many.
Privacy and security concerns	Users may feel uneasy about whether apps keep health-related data secure and private. Context sensing features create concerns about intrusion of privacy and personal safety.
Keeping control over apps	Users may feel uneasy about what apps could do without user awareness or permission. Users desire to be aware of all features and in control of all app settings.

### Smartphones as Valuable Information Sources

A commonly mentioned and uncontested use of smartphones for supporting the adoption of healthy behaviors was its ability to provide a wealth of information quickly and efficiently. Participants accessed information via the Internet regularly through their smartphones, including material relating to health. Participants described that they used their smartphones to look up potential symptoms to decide if they needed to consult a doctor, to search for healthy recipes to cook when at home, and to find advice on specific exercises they could do while at the gym. Some described a preference for multimedia information formats, for example video or audio of a fitness instructor providing guidance on an exercise. Many participants expressed interest in apps that gave information and advice that they could access “on the go”.

### Tracking Progress and Raising Awareness

Participants liked apps that provided convenient tools to help them to monitor, track, and review attempts to change or improve a health behavior.

*Most people have their phones with them most of the time, so if you’re out and about and want to check how much you’re doing or what you’re burning when you’re walking, it's a good idea.* [Hannah, Focus Group (FG) 1]

Participants who had tried apps with these sorts of features found it interesting and insightful to set and record goals, track details of progress, and as a result notice trends and patterns.

It provides a lot of kind of... it helps you keep track. The exercises you do. Because I try and set myself certain targets, do say, a certain amount of running a week, a certain amount of cycling a week.

Gareth, FG 4

Some participants considered that simply viewing records of tracked behavioral data could prompt healthier behavior. Others felt that tools to monitor or track behavior alone may be insufficient to modify behavior in the absence of some advice or intervention to help them change.

If it gave you some hints to help you out or something along those lines. Only if it had a real benefit rather than just tracking.

Leona, FG2

Participants placed importance on accuracy with regard to data tracked and recorded by apps. They were concerned about errors within the app itself, or human error (forgetting, “fooling yourself”, or “lying to yourself”) leading to inaccurate records of things like food intake, physical activity, or alcohol consumption. They were also keen that a high level of detail was preserved (eg, recording exact products/brands of foods consumed and their nutritional profiles). However, as described below, they considered entering such information burdensome and believed themselves likely either to forget to do this regularly or to give up due to boredom.

Another concern was that an app that tracks behavior and progress towards goals might reveal disappointing or upsetting results related to a behavior that could negatively affect mood, and be demotivating.

If someone is really trying to work hard on this and then it’s telling them that they have not done very well, or that they have not reached their goals then it could go either way: it could motivate them or it could just make them feel like they’re not achieving anything.

Hannah, FG1

Participants did not like the possibility that an app might explicitly “tell you off”, and were also concerned about the emotional and motivational impact of simply viewing a log that revealed large discrepancies between goals and actual achievement.

You’d probably be like, “oh well I might as well give up then, it’s too depressing”.

Amanda, FG3

### Behavior Change Apps and Social Networks

Participants appeared to perceive decisions and actions related to a healthy lifestyle to be a private activity. In general, participants viewed health apps as slightly embarrassing and being seen using one seemed socially undesirable.

You might not want the whole world to know that as it flashes up on your phone.

Aled, FG4

If this popped up, I think people would laugh at me.

Hannah, FG1

One participant had previously used a weight management app that sent reports and updates to a nominated support person. Having chosen her boyfriend to take this role, she was later embarrassed and frustrated as he continues to receive notifications of what she is *not* doing and *not* achieving.

Believe me that was a mistake, never do that! Because he still gets emails saying “it’s been 427 days since Izzy logged her calories on the program” and I’m not even using it!

Isabella, FG1

Considering apps that link and share information with social networks more generally, participants were very clear that they would not want to use app features that allow the unselective broadcasting of information about health-related goals or behaviors to friends via social media sites (eg, Facebook). Such sites were considered inappropriate places to share very personal information or to request help or support, and participants were keen to avoid presenting themselves as weak or vulnerable. Despite this aversion to apps that share data with social networks, a few specific instances where support could be harnessed in a more socially acceptable way were recognized and discussed. For example, as Isabella’s boyfriend took up running, he posted about the distances he had run on Facebook.

He really liked doing that, to share with people, and he had loads of people “liking” it, and he felt that was quite inspiring.

Isabella, FG1

Katie and others agreed that simple reports of tangible achievements were more appropriate than broadcasting health-related goals or making overt requests for emotional support. Another context where social sharing was considered acceptable was within a group of people who were working towards similar goals (eg, people using the same health app or participating in the same face-to-face intervention program). Some considered introducing an element of competition through sharing progress with others with similar goals potentially useful. Others considered tips and advice from people with similar goals to be of potential value. Participants also suggested that connection with health professionals could be useful for apps that focus on monitoring and/or changing certain health conditions.

### Scepticism Over Context Sensing

After seeing the trigger materials, participants were curious about the ability of smartphones to detect location, mood, social situation, and activity levels in order to offer specific advice and suggestions useful for that context. However, there was a strong sentiment that this technology was “gimmicky” and had little relevance to supporting healthy behavior change.

I might try it just to test it and see what it did but just to see that and see how accurate it is…Even though it’s really clever, I don’t think I’d pay attention to it. It wouldn’t make me do anything I wouldn’t normally do.

Charlotte, FG3

There was also considerable scepticism about whether phones could sense context accurately enough to be useful. Participants thought that there would be substantial potential for mistakes and agreed that erroneous sensing of mood, location, or context would be extremely irritating and could result in losing faith and interest in an app.

It’s just gonna tell you things that are wrong and you don’t need to know.

Joe, FG4

If it gets it wrong, you would automatically get really irritated by it…I think the risk of getting it wrong would be really annoying and I’d probably delete the app.

Isabella, FG1

Even if accuracy was not a problem, participants had concerns about the consequences of sensed data being used to trigger intervention components. They predicted that context-triggered advice and suggestions would produce counterproductive effects by drawing attention towards unhealthy but attractive behavior choices.

What if you were walking past [a fast food chain], you hadn’t even noticed it?... It would draw your attention to it.

Isabella, FG1

There was also concern about phones recognizing negative mood, inactivity, or lack of social contact. Participants suggested that realizing a mobile phone had recognized these states and offered just-in-time interventions could worsen your mood, or unwantedly draw attention towards feelings or situations that would otherwise be unnoticed.

The last thing you want is your phone being like “relax!”.

Lauren, FG2

Participants could also imagine many circumstances where any advice or suggestion based on sensed data would be impossible to follow and would simply be irritating.

I would get really annoyed if I was trying to do my work or something and it was telling me I hadn’t moved, I’d be like “I’m busy!”

Katie, FG1

However, some participants envisaged situations when sensed data might helpfully activate a prompt.

The amount of times I have sat watching TV or whatever when it is sunny outside, I think if [an app that prompted exercise if it sensed good weather] told me to go outside, I think that is all I would need.

Bina, FG2

If your phone knows that you’re walking then it could say “You’re walking. Why not try walking at a faster pace?” or “At the moment you are walking this fast but if you walk a little bit faster”, you know telling you things like that, by enhancing on what you’re already doing as opposed to telling you to do something you’re not.

Isabella, FG1

### Useful Prompts or Harassment?

Several participants talked about needing or wanting to be reminded of certain things, or prompted to take certain action and felt their phone could do this successfully.

I would really like it, I think I need pushing into doing anything, I’m not very motivated myself, that’s why I have all these apps to try and motivate me… I think I’m a person that responds well to little prompts, I don’t really get up and do things myself.

Ellie, FG2

Having choice in the frequency and timing of reminders/prompts was considered important, as was the tone and message content itself, with a preference towards positivity and praise.

If they can send me a message every now and then saying that I am doing well rather than just reminders. Because I quite like messages like that.

Zoe, FG2

The majority of male participants maintained that they would not find such messages from a phone supportive, with the exception of one.

If I got a message saying “you've done really well, you've done X”, I think that if I'm honest I would probably quite like it.

Aled, FG4

Despite the potential for an app to prompt helpful behavior at useful times and places, several participants discussed annoyance caused by receiving alerts, reminders, or prompts via their smartphone. They talked about their phone “nagging” or “harassing” them and described abandoning use of an app as a result.

### Motivation and Necessity of Behavior Change

An important and common theme of the discussions was that the appeal and usefulness of apps and their features depends on whether a user was *already* motivated to change lifestyle and health habits. Features such as self-monitoring tools, reminders, and prompts were often considered to be of potential use, provided that the person was committed to engaging in an effort to change and had decided to “sign up to it”, but unnecessary or irritating in the absence of pre-existing genuine motivation.

Although most participants displayed interest in making various healthy lifestyle changes, their comments suggested that they were not motivated enough to bother to consistently and “seriously” use an app to support these endeavours. Several participants discussed how behavior change apps may be most suited to people diagnosed with and being treated for either physical health or mental health problems. This included people with chronic health conditions with a higher perceived necessity for monitoring diet and exercise, ill or elderly people who might need reminding to take medications, or people engaging in psychological therapies, who might benefit from apps to help them monitor mood or complete “homework”. Participants tended to believe that they themselves were not the type of person who would want or need that level of behavior change support from a smartphone app.

The kind of people that would sign up to these are people knowing what they are signing up for and they are people who want this kind of level of support.

Isabella, FG1

If, for example, they were particularly overweight or obese and needed to lose weight for a health-related reason and needed to monitor what they were eating in that aspect then maybe they would be useful, but for me personally I don’t have any real need to use it.

Gareth, FG4

### Necessity for Efficiency and Convenience

Participants described how smartphone apps were most appealing and useful when they were well integrated with how they used their phones naturally. Participants disliked apps that drain battery power, take up excessive space/memory, or cannot run in the background without affecting other phone functions.

Participants spoke about their smartphones as being valuable because they make things quicker, easier, and more pleasurable to do. However, many health-related apps that they have tried or heard about were time-consuming, burdensome, or even stressful to set up and interact with. Participants displayed very little patience for using these apps.

It’s quite easy to lose interest really because it is quite an effort and, like you said, nobody wants to spend all their life writing down what they want on their phone.

Aled, FG4

After about a week I was just forgetting to do it and just, this is too much hassle.

Gareth, FG2

### Disposability

The need for both appealing features and very low burden became particularly clear as participants described how they choose to use or discontinue use of apps**.** Given that there is plenty of choice of free apps, participants were not particularly committed to any individual app and might try out several that seem appealing or that they are curious about. Participants were very clear that apps were easily discarded if they did not meet expectations. Interest in apps appeared to be fleeting and participants did not expect to use them on a regular or long-term basis.

Sometimes if you download them and they are not as good as you thought they’d be then it’s a bit rubbish, but they’re free so it doesn’t really matter.

Jessica, FG3

### Credibility and Accuracy

Participants expressed concern about whether apps they used were from reputable and legitimate sources. Apps developed by experts were considered preferable and more persuasive than those from unknown or less reputable sources.

If you knew that the app was being controlled or something by some kind of doctor it might make you listen more, rather than just anyone could be sitting at a computer and writing these things.

Marie, FG3

Participants were also concerned about whether information and advice given was safe and accurate. Several had encountered apps that they believed had provided inconsistent or inappropriate advice, which had caused them to abandon use.

### Privacy and Security Concerns

Participants voiced a number of apprehensions related to personal privacy and security. Many were uneasy about apps being able to keep their health data secure and private. Some preferred password access, but several participants also complained about the effort involved in creating accounts and entering passwords and suggested that this may dissuade them from using an app.

A key worry was whether data entered or sensed by the smartphone would get into the hands of third parties, including the app developer and other companies. They were particularly sensitive about physical and mental health data being used to tailor advertisements to them.

Participants were also uneasy about some of the context-sensing capabilities of smartphones, describing them as creepy and intrusive, and many suggested these features would discourage their use of an app.

I’d be wondering if it could actually hear your conversation, you’d have no sense of privacy.

Marie, FG3

Despite some concerns, there were participants who were very relaxed about how their data might be used by an app for advertising. Anthony described himself as “desensitized” to how the Internet uses data about you while Aled was unconcerned, believing that his physical and mental health were not interesting enough to be monitored by third parties. Sensing of location, however, prompted considerable discussion amongst female participants about personal safety (specifically, stalkers and burglars seeking opportunistic moments using GPS data to establish that someone was away from home).

### Keeping Control Over Apps

Most participants had experienced both smartphone apps and websites (especially online social networking sites) behaving in unexpected and undesirable ways. Participants were uneasy about what smartphones might do without their awareness or permission and its consequences given the personal and sensitive nature of health information.

You forget that it’s connected to the Internet and that it can do anything with it, imagine if it just puts [information about your health] up on your Facebook.

Isabella, FG1

Some participants mentioned they would want to be made fully aware of what the app would do prior to using it. However, participants also said they were unlikely to want to read lengthy instructions or terms and conditions.

Participants also wanted to be in control of settings and to personalize the app during setup (and review and edit settings later on) depending on what suited them.

You want to be the one who sets up what it’s going to do.

Leona, FG2

Some participants talked about wanting to be the ones that initiated interaction with apps at convenient times for them, rather than the phone alerting, or prompting them to use it.

If I wanted to hear from the app, I’d go into the app rather than it just pop up.

Ellie, FG2

Participants also discussed wanting the ability to turn off features of the app (eg, reminders or prompts) or indeed silence or disable the entire app at certain times when behavior change was not considered a priority.

Something that you could turn on and off rather than having something that’s constantly in your face.

Leona, FG2

## Discussion

### Principal Findings and Questions for Future Investigations

This study explored young adults’ experiences and views about health-related smartphone apps, including how they perceive various features and their willingness to use these apps. In overall concordance with the limited pre-existing research in this area [1-8], our participants displayed muted enthusiasm about the role of smartphone apps in assisting with health-related behavior change, some positive experiences of using such apps, and tentative willingness to try apps, even though many of them did not perceive a pressing need to change their health habits. The focus group data suggested a number of features of apparent importance and interest to young people. In Textbox 1 these are depicted as a preliminary checklist of app features and characteristics that app developers may wish to consider when creating health behavior apps for this population.

The findings from this study that we consider to be particularly interesting and novel relate to the apathy, concerns, and frustrations around health apps. The remainder of this discussion therefore focuses on these issues. We present 5 major challenges identified from the present study that appear to need further consideration and research in order to inform the development of acceptable and effective behavior change apps (Textbox 2).

**A preliminary checklist of valuable features of health behaviors apps.**

Low effort and pleasant to use
Sustaining interest over long periods of time
Cost and effort free to download and set up
Developed by legitimate experts and the developer’s credentials made explicit
Includes features to help users track health-related behavior, including setting and monitoring goals
Provides feedback and advice that guide people in how they can change behavior
Generates positively framed alerts and reminders that are relevant and timely but not too frequent
Easily turned off or disabled (certain settings and the entire app)
Accurate and reliable information and tracking functions
Discrete and with adequate privacy settings
Use of the app does not negatively impact or restrict any other uses of the smartphone
Clarity about what app will do—no surprises

**Challenges for acceptable and effective behavior change apps.**

(How) Can we keep people using behavior change apps for an extended period of time?
(How) Can we give users features that are desirable and effective without requiring unacceptable levels of effort?
(How) Can we provide accurate and timely information, feedback, and advice without adverse effects on mood?
(How) Can we harness context sensing in a way that users feel comfortable with, trust, and find useful?
(How) Can we harness social media to make interventions engaging and provide social support in a way that users are willing to engage with?

#### Challenge 1: (How) Can We Keep People Using Behavior Change Apps for an Extended Period of Time?

An important insight from the current study was that participants lacked commitment to using any particular app and seemed likely to engage in only transient, casual use. This finding is of enormous relevance to apps that aim to support long term behavior change (eg, improving diet or physical activity levels to manage weight). This study raises doubts around whether users will use behavior change apps for long periods of time, a critical issue that will affect the effectiveness of many behavior change apps. Future research will need to examine duration of use and features that influence this. From this research, 2 factors that might be relevant to discontinued use of an app were the effort required to use the app and emotional responses to the app. These are discussed further below.

#### Challenge 2: (How) Can We Give Users Features That Are Desirable and Effective Without Requiring Unacceptable Levels of Effort?

An important and encouraging finding from this research was that many participants were positive about using smartphones to track behavior, set targets, review progress, and receive graphical or verbal commentary on success. Previous research on the views of healthy adults as well as at-risk and chronically ill participants also suggested that monitoring and tracking features are acceptable and valuable [1,3,4,9]. Encouragingly, these features also map onto key behavior change techniques that behavioral science research has established as effective for supporting behavior change, namely self-monitoring, goal-setting, and receiving feedback on performance [10,11]. Importantly, this study suggested that although these features were attractive to users in principle, they might prove to be overly burdensome. Our participants were keen for their behavior recorded accurately and in detail, yet did not want to enter this information regularly. Furthermore, they predicted that they were likely to forget to monitor and track, yet would be irritated by prompts and reminders and would ignore them. In addition, they wanted to understand the features of an app and not be concerned that it would do something unexpected, yet they were unwilling to spend time reading explanations or instructions.

Previous research on smartphone apps in general suggested that simplicity, efficiency, and pleasure influences continued use [27]. When considered in conjunction with the low commitment that characterized the participants in this study, features that were perceived as effortful and burdensome were likely to influence use negatively. The challenge for future research is to establish whether it is possible to incorporate attractive and effective behavior change techniques into phone apps while maintaining a low user burden.

#### Challenge 3: (How) Can We Provide Accurate and Timely Information, Feedback, and Advice Without Adverse Effects on Mood?

Another key issue that the current study exposed was the potential for interactions with health behavior apps to trigger negative emotional reactions. Participants described becoming annoyed with receiving prompts, alerts, reminders, and messages. They described irritation or disappointment as a consequence of inaccurate, untimely, or irrelevant notifications or advice. They described becoming upset or demotivated by viewing logs or records that showed they were not succeeding in meeting a goal, and by feedback with a punitive or didactic tone. They also predicted heightened distress in response to app-induced awareness of health-related states. Importantly, participants suggested that these reactions might lead them to discontinue app use. Negative emotional responses to apps would benefit from more detailed investigation in a naturalistic setting to ascertain whether they are widespread and whether they are indeed related to app discontinuation. A challenge will be to discover whether there are optimal ways that apps can communicate with the user to engage them in behavior change, keep them aware of their progress towards goals, and provide relevant and timely advice and support without generating adverse emotional reactions and threatening adherence.

#### Challenge 4: (How) Can We Harness Context Sensing in a Way That Users Feel Comfortable With, Trust, and Find Useful?

To our knowledge, this study was the first to report potential users’ views on the use of context sensing to support timely and appropriate behavioral interventions for health. Computer scientists and behavior change experts have been enthusiastic about developments in context sensing, considering it as a potential solution to the problem that self-monitoring is burdensome and unappealing for users, and seeing it as a way of encouraging timely engagement with digital interventions. In contrast, the views of the study sample were characterized by scepticism and concern. It seems that several substantial issues need considerable research and attention if context sensing is to be applied in a way that users perceive as useful and acceptable.

First, the study showed that participants lacked faith in the accuracy with which a smartphone could sense relevant states (eg, mood, activity levels) and expected that incorrect and irritating suggestions would make them mistrust the app and cease using it. Given such low expectations and the potentially adverse consequences of losing a users’ trust in an app, demonstrating consistent accuracy of context sensing and that it has face validity may be an important precursor to using it to trigger advice and intervention within health apps. Second, participants predicted that even accurate sensing might produce counter-productive emotional and behavioral consequences. Future research should include a careful monitoring of unanticipated effects from sensing-driven interventions and advice. Third, in order to promote willingness to use context-sensing apps for sensitive topics such as health, careful consideration of potential users’ security and privacy concerns is necessary. This might include establishing whether the risks they anticipate are realistic, possible to eliminate or reduce, and how information about security and privacy is communicated to users.

Although this study exposed mostly negative attitudes towards context sensing it did suggest opportunities to use this technology in ways that were more interesting and acceptable. Specifically, using sensing to recognize opportunities and successes such as establishing when somebody is in a receptive mood, suitable location, and/or already engaging in the target behavior may be worthwhile to pursue. Overall, careful exploration of if and how health behavior change apps can use context sensing in a way that users perceive as acceptable and useful is now needed.

#### Challenge 5: (How) Can We Harness Social Media to Make Interventions Engaging and Provide Social Support in a Way That Users Are Willing to Engage With?

It has been proposed that social networking media may facilitate social support for behavior change and keep people interested and engaged in digital interventions [12]. However, a key finding from this study was a disinclination to use health apps that linked to online social networks. Our participants described an aversion to involving existing online social contacts in their efforts to change behavior and considered doing so to be socially unacceptable, except under certain circumscribed conditions. Previous research on views about this has been mixed. Obtaining real time social support from digital networks was a key benefit of mobile interventions cited by overweight/sedentary pre-diabetic patients [13]. Other research has highlighted how participants in a physical activity intervention had positive experiences of sharing progress and competitiveness using their mobile device [28] and how participants were interested in and enjoyed recording and sharing emotional states and participating in socially supportive activities via a mobile app [29]. Conversely, other studies suggested more negative attitudes, that users of an app targeted to reduce sedentary behavior were not interested in sharing their progress with their social networks [30], and users of apps to increase physical activity enjoyed sharing with social networks when they were being active but were uncomfortable doing so when they were not achieving high levels [31].

Given the large body of evidence implicating social support in successful behavior change, the scope for online social networks to be harnessed in behavior change apps deserves further detailed examination. Specifically, attempts to better understand the conditions under which it is acceptable and useful to link health smartphone apps to online social networks and the most acceptable and effective ways to facilitate and encourage support from these networks would be beneficial.

### Study Limitations and Conclusion

Several key limitations influence the strength of the conclusions and recommendations drawn from the current study. First, the sample was small, self-selected, and drawn from a university population. This allowed, as intended, a detailed exploration of the views of an age group who have not been the focus of extant health app research yet are likely to own and use smartphones [2] and are likely to have scope for improving behaviors such as diet, physical activity, and alcohol consumption. However, the sample may differ from other young people and the extent to which the themes discerned here are applicable across other populations is unclear. Another limitation of this study was that the views and experiences it uncovered were either retrospective accounts of experiences with apps or discussions of *hypothetical* app use. Neither are necessarily accurate portrayals of how apps are actually used and the affective, cognitive, and behavioral responses that they generate. Further research could establish whether findings emerging in this study are observed when actual app use is studied. Useful data collection methods might include phone-generated records of the nature of actual app use, and quantitative and qualitative data on changes in behavior, emotions, and thoughts measured frequently and repeatedly as people use apps to support behavior change.

Given the limitations of the current study, its findings serve as research questions for future investigations, rather than definitive solutions to developing effective health apps. This study presents important messages to developers and behavioral scientists involved in creating smartphone apps that aim to support long-term changes to health-related behavior. A number of unresolved challenges remain, threatening efforts to develop apps that are effective in changing behavior to improve health. We recommend further investigation to resolve issues with lack of long-term use and commitment. Efforts should be made to explore how appealing and effective behavior change techniques can be incorporated into health behavior change apps while avoiding content, features, and technologies that embarrass, irritate, upset, worry, or burden users.

## Multimedia Appendix 1

Interview schedule.

## Multimedia Appendix 2

Trigger materials.

## Abbreviations

FG: focus group
SMS: short message service
