# Supplementary material for: Opportunities and Challenges for Smartphone Applications in Supporting Health Behavior Change: Qualitative Study
Source: J Med Internet Res. 2013 Apr 18;15(4):e86. doi: 10.2196/jmir.2583 (PMC3636318; doi:10.2196/jmir.2583)
Supplement: Supplementary file 1 [file jmir_v15i4e86_app1.pdf]

Appendix 1: Focus group schedule

Section 1

- Can we start by asking about whether you have had any experience of using your mobile phones for anything to do with health?
- Can you tell me about anything you've found useful to support making changes to behaviour (e.g. eating, smoking, drinking alcohol)?
- What sorts of things make you want to use it/stop using it?
- To non-users is this technology something you knew about? Interested in? Can you tell me why/why not?

Section 2.

[Following first set of trigger materials]

- What do you think/how do you feel about these?
- Is there anything about this you think would be useful for you?
- Could you tell me about whether you would be interested in using it? Could you explain more about how and when you might use it?
- What else would you like it to do?
- What about any problems or concerns you can see with this?

Section 3.

[Following second set of trigger materials]

- What do you think/how do you feel about these?
- Is there anything about this you think would be useful for you?
- Could you tell me about whether you would be interested in using it? Could you explain more about how and when you might use it?
- What else would you like it to do?

- What about any problems or concerns you can see with this?
