# Supplementary material for: Opportunities and Challenges for Smartphone Applications in Supporting Health Behavior Change: Qualitative Study
Source: J Med Internet Res. 2013 Apr 18;15(4):e86. doi: 10.2196/jmir.2583 (PMC3636318; doi:10.2196/jmir.2583)
Supplement: Supplementary file 2 [file jmir_v15i4e86_app2.pdf]

## Appendix 2: Trigger materials

### Trigger materials: set 1

“Next, I’m going to show you some slides about some of the features of health apps that are available at the moment. I realise that some of these have already been raised in your discussions. I have some printouts of these which I’ll hand out in a second so that you can look at these as we continue our discussion.”

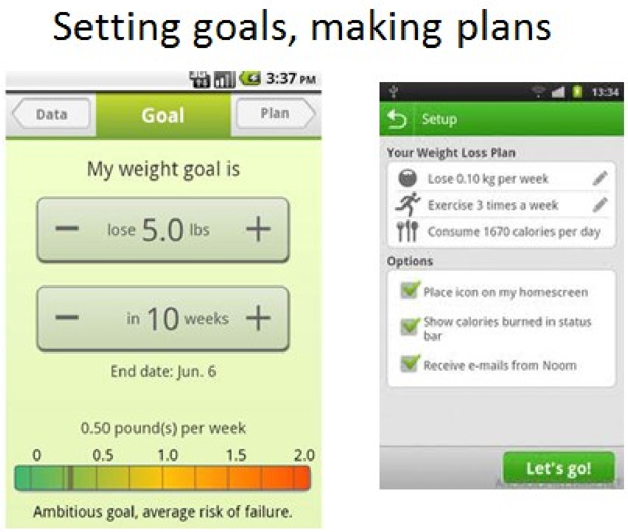


“Some of the key features of health apps that are available at the moment are that they can help you to set goals or plans. These pictures are apps aimed at weight management”

1. CardioTrainer http://www.noom.com/cardiotrainer/about.php
2. Noom Weight Loss Coach http://www.noom.com/products/

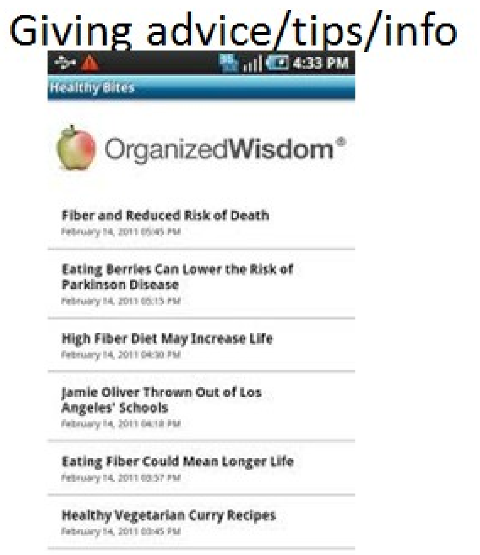


“Apps often provide access to information, advice or tips. Here is an example. This one is about healthy eating and nutrition”

1. Healthy bites, Organized Wisdom. http://www.organizedwisdom.com/Home

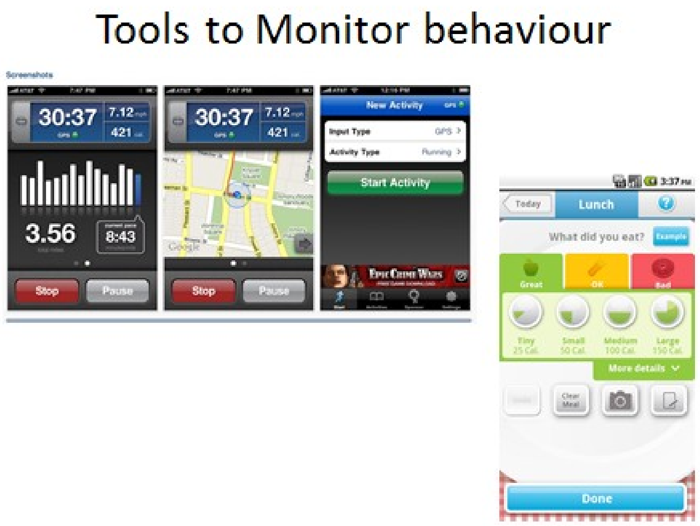


“Apps often provide ways of tracking things to do with health behaviour.  Again, these pictures are of apps related to weight and fitness management. Lots of apps include things like calorie counters, food logs and exercise logs. Some can automatically get input from a device external to your phone like a pedometer or internal to your phone like an accelerometer. So they can do things like work out your distance, calories burned and so on”

1. Noom Weight Loss Coach http://www.noom.com/products/
2. Runkeeper http://runkeeper.com/running-app

## Tools to monitor mood and wellbeing

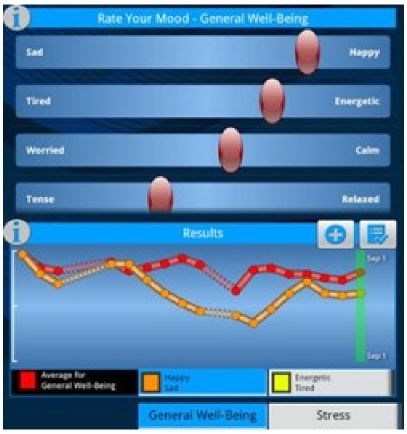


“Apps can also provide ways of tracking or monitoring things link stress levels, mood and energy levels. You can log or rate these things on the phone. The phone allows you to look at your data, for example this picture shows an app which displays mood in a graph”.

1. T2 mood tracker http://t2health.org/apps/t2-mood-tracker

## Reminders

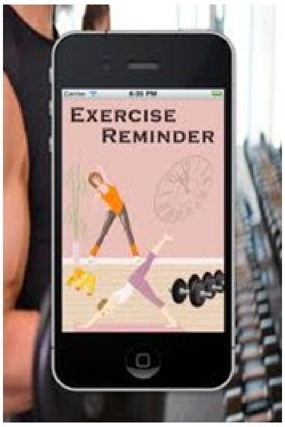


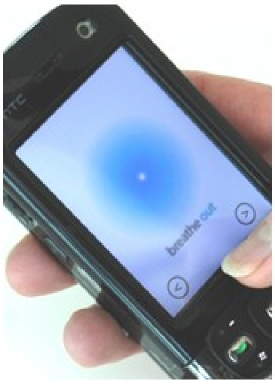


“Phones can beep or vibrate or come up with a message or notification at preset times during the day. The lefthand picture shows an app that gives you a reminder to do some exercise

Apps could also do more than just remind you- they can run a little programme to give you some coaching; for example the picture on the right is of a smartphone running a programme that gives the user instructions on how to do some sort of relaxation exercise based on breathing techniques”.

1. Exercise reminder HD lite https://itunes.apple.com/gb/app/exercise-reminder-hd-lite/id463200694?mt=8
2. Breathing visualisation app , Morris M, Kathawala Q, Leen T, Gorenstein E, Guilak F, Labhard M, Deleeuw W. Mobile Therapy: Case Study Evaluations of a Cell Phone Application for Emotional Self-Awareness. JMIR 2010 Apr; 12(2); e10

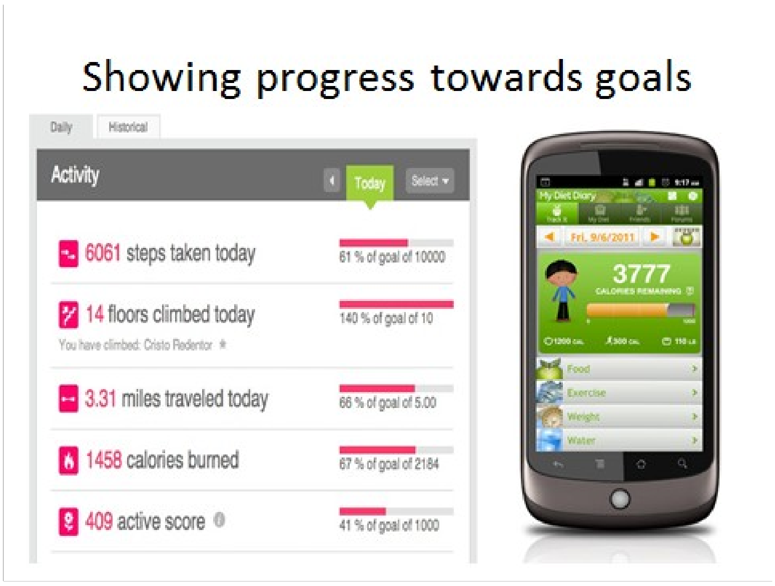


“Apps can show you how you’ve been getting on with health-related goals.

This is often in some sort of visual format as you can see in these two pictures. For example progress towards goals can be shown as a progress bar, or as a percentage, or visually as colours; green for good, yellow for OK, red for not doing so well.”

1. Fitbit http://www.fitbit.com/uk
2. My diet diary https://play.google.com/store/apps/details?id=org.medhelp.mydiet&hl=en

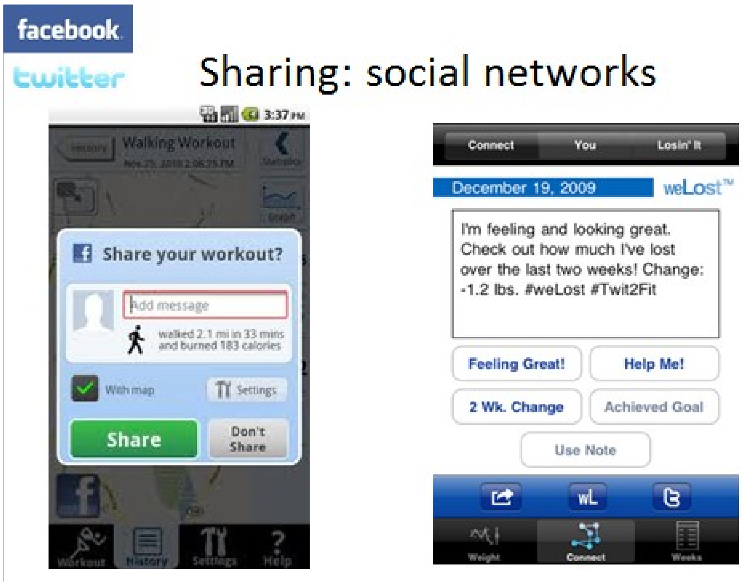


"Apps often have the option to share what you are doing with your online social networks for example facebook and twitter. For example, the one on the left offers the option to be able to post things to facebook. For example it can tell your friends on facebook about the exercise you've just done:  how much, how far, how many calories.

The app on the right allows you to share how things are going for you- good or bad. So it encourages sharing success and also asking for help and support."

CardioTrainer http://www.noom.com/cardiotrainer/about.php

We Lost http://welosttogether.com

**Trigger materials: set 2**

"Next, I'm going to show you some slides about some of the newer features that health apps are starting to include, or that they might start using in the near future.  Again, I have done some printouts for you to look at in a minute as we continue talking."

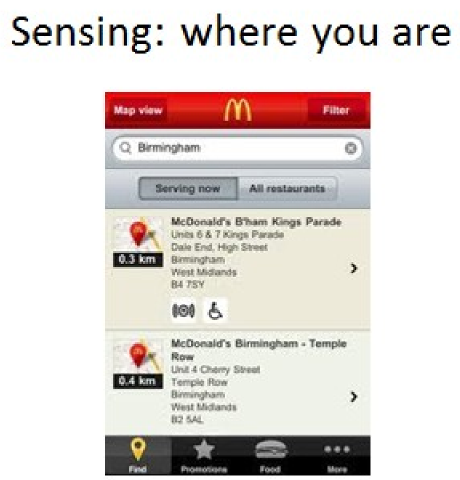


“GPS (location) sensors in your phone can prompt your phone to do something based on your location. So, you may already have experience of companies offering you products and services nearby to where you are. Or social media apps may tell you who is in the vicinity and prompt connections with other people. Health apps may be able to use data about your location in different ways

They may be able to notice that you are in a risky/vulnerable position in terms of making good health choices.  Apps could be developed where location triggers something on the phone to dissuade you from making unhealthy choices, to give you tips on how to boost your willpower and support healthy choices.

Apps could also use GPS data to sense opportunities for healthy behaviours. For example they might sense proximity to green space, leisure facilities and make suggestions about walking, sports etc”.

1. Macdonalds restaurant finder http://appfinder.lisisoft.com/app/mcdonalds-restaurant-finder.html

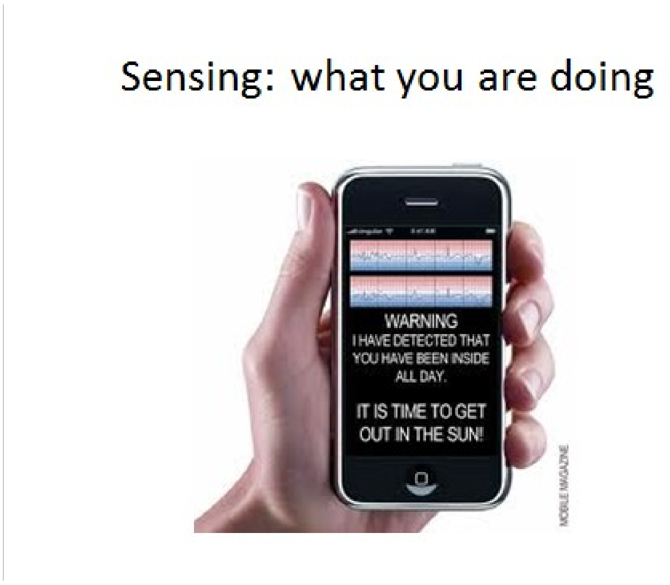


"Technology is getting to the stage where phones are clever enough to combine lots of sources of data to 'sense' what you are doing. They might use GPS, accelerometer (whether phone has been moving around) time of day and other bits of data to determine where you are and what you are doing. Based on this some specific advice or reminders might be triggered to appear on your phone.

So in this example, the phone has 'guessed' that the user hasn't been out and about and is offering a suggestion based on this."

1. http://www.mobilemag.com/2012/02/13/future-apps-will-help-detect-mood-and-prevent-depression/

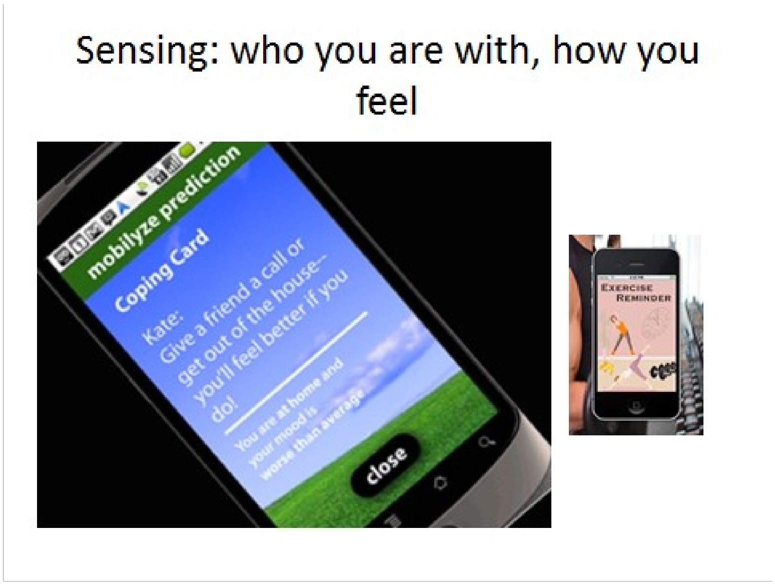


"It looks like smartphones will soon be able to use lots of different sensors and data sources to work out who you are with and how you are feeling. For example GPS sensors can determine if

you are at home or at work, they can combine this with information about time of day, whether it is weekday/weekend, presence of other phones nearby, whether you've recently received/made calls or texts, even the tone and volume of your voice. From this phones will be able to make a prediction about what your mood is like: whether you are in a good mood, stressed, feeling down. It can also work out whether you have been sociable and guess at whether you might be lonely.

Based on this it might offer specific advice or information or give you something to do on the phone to help with that specific situation.

If apps can guess at what your mood is like and whether you are with people or alone, then apps might use this sort of information to trigger reminders. So, for example this exercise reminder that was in an earlier slide might be activated by how you are feeling, rather than at a pre-determined time of day".


1. **'Mobilyse' Burns M, Begale M, Duffecy J, Gergle D, Karr C, Giangrande E, Mohr D, Harnessing Context Sensing to Develop a Mobile Intervention for Depression. JMIR 2011 Jul,13(3): e55**
2. Exercise reminder HD lite https://itunes.apple.com/gb/app/exercise-reminder-hd-lite/id463200694?mt=8
